# Distinct Network Morphologies from In Situ Polymerization of Microtubules in Giant Polymer‐Lipid Hybrid Vesicles

**DOI:** 10.1002/adbi.202400601

**Published:** 2025-03-12

**Authors:** Paula De Dios Andres, Mousumi Akter, Cecilie Ryberg, Brigitte Städler, Allen P. Liu

**Affiliations:** ^1^ Interdisciplinary Nanoscience Center (iNANO) Aarhus University Aarhus 8000 Denmark; ^2^ Department of Mechanical Engineering University of Michigan Ann Arbor Michigan 48109 USA

**Keywords:** artificial cells, giant hybrid vesicles, microtubules, tubulin polymerization

## Abstract

Creating artificial cells with a dynamic cytoskeleton, akin to those in living cells, is a major goal in bottom‐up synthetic biology. In this study, we demonstrate the in situ polymerization of microtubules encapsulated in giant polymer‐lipid hybrid vesicles (GHVs) composed of 1,2‐dioleoyl‐*sn*‐glycero‐3‐phosphocholine and an amphiphilic block copolymer. The block copolymer is comprised of poly(cholesteryl methacrylate‐*co*‐butyl methacrylate) as the hydrophobic block and either poly(6‐O‐methacryloyl‐D‐galactopyranose) or poly(carboxyethyl acrylate) as the hydrophilic extension. Depending on the concentrations of guanosine triphosphate (GTP) or its slowly hydrolyzable analog, guanosine‐5′‐[(α,β)‐methyleno]triphosphate (GMPCPP), different microtubule morphologies are observed, including encapsulated microtubule networks, spike protrusions, as well as membrane‐associated or aggregated microtubules. Overall, this work represents a step forward in mimicking the cellular cytoskeletons and uncovering the influence of membrane composition on microtubule morphologies.

## Graphical abstract

The successful in situ polymerization of microtubules in giant polymer‐lipid hybrid vesicles is illustrated. The microtubules formed in the giant hybrid vesicles exhibit different morphologies including lumenal network formation and membrane association.

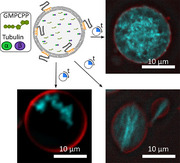

## 1 Introduction

The design and assembly of artificial cells – life‐like units that mimic their natural counterparts – represent a core focus of bottom‐up synthetic biology. Key features of mammalian cells, such as energy generation,^[^ ^1^ ^]^ growth and division,^[^ ^2^ ^]^ and cellular communication,^[^ ^3^ ^]^ are essential considerations in engineering these artificial systems, as recently summarized in detail.^[^ ^4^ ^]^

Artificial cells have been constructed from a variety of scaffold‐forming building blocks.^[^ ^4a^ ^]^ Traditionally, lipid‐based vesicles, such as liposomes or giant unilamellar vesicles (GUVs), have been employed as primary scaffolds to replicate cellular compartments. However, the plasma membrane in natural cells consists of more than just lipids; it also includes cholesterol, transmembrane and peripheral proteins, as well as glycoproteins and glycolipids. Consequently, researchers have begun exploring polymer‐lipid hybrid vesicles to incorporate this increased complexity, advancing the assembly of plasma membrane mimetics as detailed in a recent review.^[^ ^5^ ^]^

The shape of a cell is governed by its cytoskeleton, a dynamic network composed of actin filaments, microtubules, and intermediate filaments. Reconstituting this cytoskeletal network in artificial cells remains challenging as reviewed by Bashirzadeh et al.,^[^ ^6^ ^]^ Koenderink and coworkers^[^ ^7^ ^]^ and Ganar et al.^[^ ^8^ ^]^. Recent studies have encapsulated actin within GUVs to investigate how actin network architecture influences membrane deformation.^[^ ^9^ ^]^ Additionally, the role of microtubules in membrane shaping has been extensively researched. For example, Sato et al. designed a shape‐changing molecular robot that consisted of a vesicle encapsulating microtubules and the motor protein kinesin.^[^ ^10^ ^]^ This system enabled continuous vesicle shape changes driven by the gliding motion of kinesins on microtubules tethered to the vesicle's inner membrane. Other approaches have included the encapsulation of tubulin into GUVs followed by the in situ polymerization into microtubules. Notably, GUVs containing tubulin and guanosine triphosphate (GTP) transitioned from spherical shapes to straight‐line protrusions post‐polymerization in the presence of different microtubule‐associated proteins.^[^ ^11^ ^]^ Furthermore, Hayashi et al. demonstrated controlled polymerization and depolymerization of microtubules within GUVs by applying and releasing hydrostatic pressure, contributing toward the development of a motile artificial cell.^[^ ^12^ ^]^ Weiss et al. developed a strategy for pico‐injecting biomolecules into pre‐formed empty GUVs and demonstrated in situ microtubule polymerization by pico‐injecting tubulin and GTP into GUVs.^[^ ^13^ ^]^ Gavriljuk et al. recently engineered artificial cells capable of converting extracellular signals into cytoskeletal changes.^[^ ^14^ ^]^ They encapsulated centrosomes together with tubulin and GTP to obtain a dynamic microtubule aster system. The microtubules could deform the GUV membrane with spiking protrusions under specific conditions. Additionally, a signal actuation system based on light‐induced stathmin‐kinase translocation was encapsulated leading to membrane deformations due to stathmin‐dependent microtubule growth near the membrane. An intriguing example was reported by Bermudez et al., in which a synthetic cortex pattern of polymerized microtubules was reconstituted into GUVs in the presence of GTP or guanosine‐5′‐[(α,β)‐methyleno]triphosphate (GMPCPP, a slowly‐hydrolyzable GTP analog), anchored by bait proteins, resulting in large‐scale deformations and reshaping of the compartment.^[^ ^15^ ^]^ To our knowledge, this is the only reported example of cortex assembly occuring without microtubules in the GUV lumen. A notable study was reported by Kattan et al., who employed a cell‐free expression system to express tubulins BtubA and BtubB, which assembled into microtubules in the presence of GTP, deforming membranes without the need for external stimuli or microtubule‐associated proteins.^[^ ^16^ ^]^

**Scheme 1:**
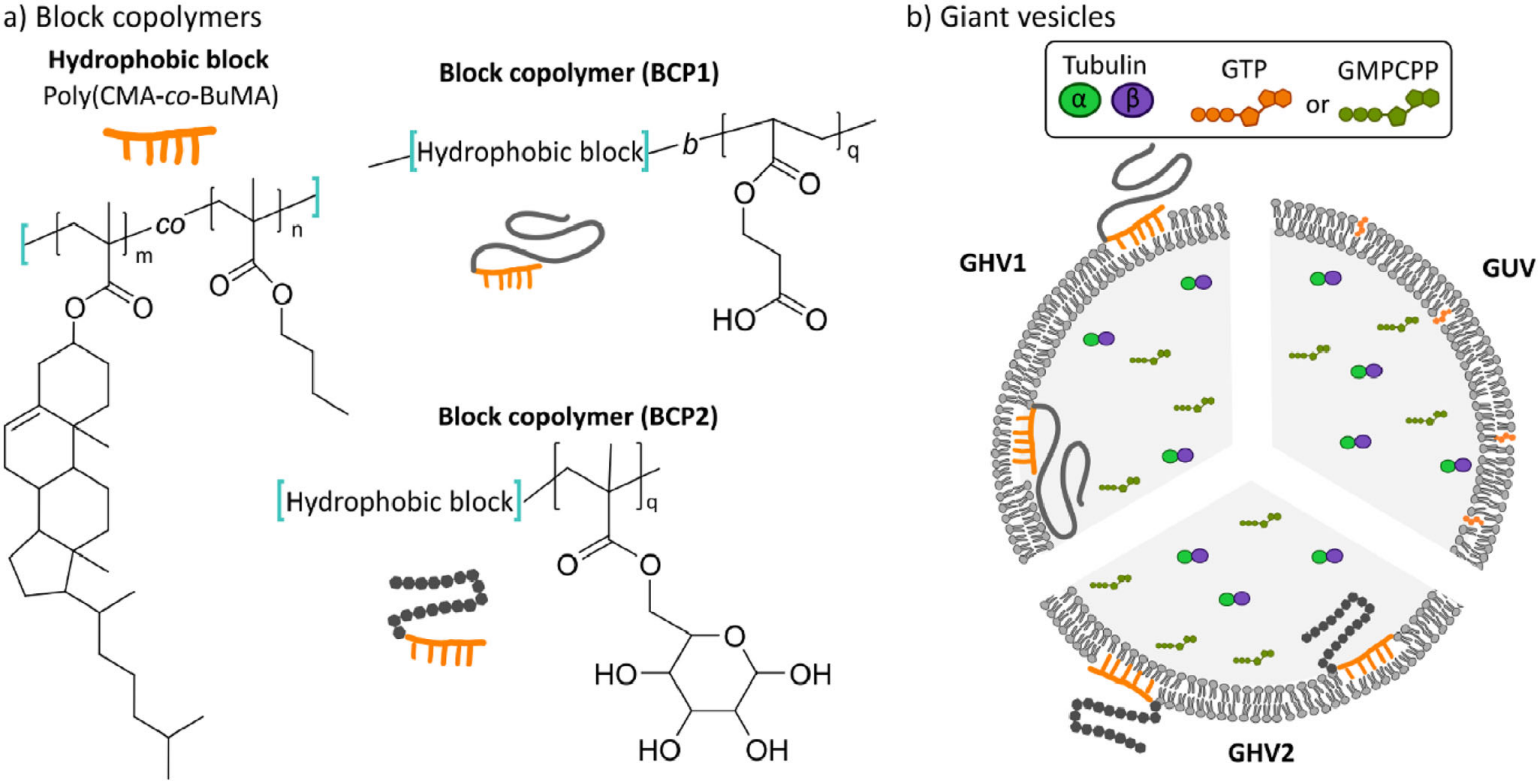
a) The molecular structures of the amphiphilic block copolymers used for the assembly of GHVs. i) The hydrophobic block poly(cholesteryl methacrylate‐*co*‐butyl methacrylate) and the resulting amphiphilic block copolymers BCP1 (poly(cholesteryl methacrylate‐*co*‐butyl methacrylate)‐*block*‐poly(carboxyethyl acrylate)) and BCP2 (poly(cholesteryl methacrylate‐*co*‐butyl methacrylate)‐*block*‐poly(6‐O‐methacryloyl‐D‐galactopyranose)) after the extension with either 6‐O‐methacryloyl‐D‐galactopyranose or carboxyethyl acrylate. b) A cartoon illustrating the different types of assembled giant vesicles, (i) GUV, constructed from 1,2‐dioleoyl‐*sn*‐glycero‐3‐phosphocholine (DOPC) and cholesterol, (ii) GHV1, from DOPC with BCP1 and (iii) GHV2, from DOPC and BCP2, all encapsulating tubulin with GTP or GMPCPP.

Here, we aimed to explore the characteristics of in situ encapsulated microtubule formation in giant vesicles in relation to membrane composition and nucleotides. Specifically, i) we synthesized two amphiphilic block copolymers with poly(cholesteryl methacrylate‐*co*‐butyl methacrylate) as the hydrophobic block and either poly(carboxyethyl acrylate) or poly(6‐O‐methacryloyl‐D‐galactopyranose) as the hydrophilic extension to be used for the assembly of giant hybrid vesicles (GHVs) together with 1,2‐dioleoyl‐*sn*‐glycero‐3‐phosphocholine (DOPC) (**Scheme** **1**a), and ii) we compared the in situ polymerization of encapsulated tubulin in the different GHVs in the presence of either GTP or GMPCPP (Scheme 1b).

## 2 Results and Discussion

### 2.1 Polymer Synthesis

Polymer‐lipid hybrid vesicles (HVs) are promising alternatives to liposomes and polymersomes, combining selected features from both to offer enhanced functionality, as we recently reviewed.^[^ ^5^ ^]^ Most HVs are constructed using an amphiphilic block copolymer featuring poly(ethylene glycol) (PEG) or poly(ethyl oxide) (PEO) as the hydrophilic block and poly(1,2‐butadiene) (PBd), poly(isobutylene) (PiB), poly(dimethyl siloxane) (PDMS) or poly(cholesterol methacrylate) (PCMA) as the hydrophobic counterpart. Among these, PCMA stands out for its nature‐like properties due to the presence of the cholesterol groups. Specifically, we have previously demonstrated that the hydrophobic block poly(cholesteryl methacrylate‐*co*‐butyl methacrylate) (p(CMA‐*co*‐BuMA)) in amphiphilic block copolymers was an excellent choice for hybrid vesicle assembly.^[^ ^17^ ^]^ Consequently, we synthesized p(CMA‐*co*‐BuMA) with 4.7 kDa molecular weight, which was extended with carboxyethyl acrylate to obtain BCP1 (M_n_ = 15 kDa) following a previously established procedure.^[^ ^17^ ^]^

**Figure 1:**
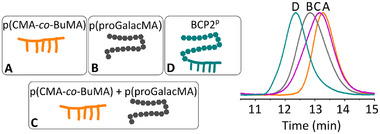
GPC traces of the homopolymers p(CMA‐*co*‐BuMA) (A, orange) and p(proGalacMA) (B, gray) as well as their mixture (C, pink) and BCP2^p^ (D, turquoise).

In addition, we synthesized the sugar‐like monomer 6‐O‐methacryloyl‐1,2:3,4‐di‐O‐isopropylidine‐α‐galactopyranose (proGalacMA) according to previous methods (Scheme S1 and Figure S1a, Supporting Information).^[^ ^18^ ^]^ This monomer was then used to extend p(CMA‐*co*‐BuMA) by reversible‐addition fragmentation chain transfer (RAFT) polymerization yielding BCP2^P^ (the amphiphilic block copolymer with protected sugars, Scheme S2, Supporting Information). The resulting hydrophilic extension imitated the plasma membrane more closely due to its sugar‐like nature compared to the previously reported alternatives. The as‐synthesized BCP2^P^ had the alcohol groups on the hydrophilic extension protected with acetal groups, which meant that there was no amphiphilicity yet. Gel permeation chromatography (GPC) was used to confirm that BCP2^P^ was indeed a block copolymer (**Figure** **1**). The homopolymers p(CMA‐*co*‐BuMA) and p(proGalacMA) as well as their mixture were used for comparison. The GPC traces of the homopolymers p(CMA‐*co*‐BuMA) and p(proGalacMA) showed a single peak eluting at ≈13.3 and ≈12.8 min, respectively, suggesting that p(proGalacMA) had higher molecular weight than p(CMA‐*co*‐BuMA) due to the shorter retention time. In addition, when considering the GPC traces of a mixture of the homopolymers p(CMA‐*co*‐BuMA) and p(proGalacMA), a single broad peak, eluting at ≈13.2 min, was observed. The peak was slightly skewed toward higher molecular weights, suggesting the presence of both homopolymers, but they were too similar in molecular weights to be separated in our GPC setup. Importantly, GPC trace for BCP2^P^ showed a single peak eluting at ≈12.3 min. In other words, the shorter retention time for BCP2^P^ strongly supports the presence of a connected block copolymer with higher molecular weight than the individual blocks.

Additionally, we used ^1^H NMR to confirm the synthesis of BCP2^P^ (Figure S1b and Scheme S2, Supporting Information). From the ^1^H NMR, a degree of polymerization of 30 was determined, resulting in an effective molecular weight of M_n_ = 14.6 kDa for BCP2^P^.

Finally, the alcohols in BCP2^P^ were deprotected with trifluoroacetic acid (TFA) to remove the acetal groups, and the amphiphilic p(CMA‐*co*‐BuMA)‐*b*‐p(GalacMA) (BCP2) was confirmed by ^1^H NMR (loss of peaks *c* at 1.29–1.52 ppm; Scheme S3 and Figure S2, Supporting Information). Furthermore, a decreased solubility of BCP2 was observed, i.e., BCP2 was insoluble in a variety of typical solvents (e.g., chloroform or tetrahydrofuran (THF)), except pyridine. We speculated that this solubility issue might originate from the high level of amphiphilicity of BCP2. Finally, we conjugated 4‐(N‐chloroformylmethyl‐N‐methylamino)‐7‐nitro‐2,1,3‐benzoxadiazole (NBD‐COCl) to BCP2, resulting in the fluorescent version BCP2^f^.

### 2.2 Giant Hybrid Vesicle (GHV) Characterization

GHVs were assembled using a modified continuous droplet interface crossing encapsulation (cDICE) method^[^ ^19^ ^]^ to incorporate the block copolymers into the bilayer of 1,2‐dioleoyl‐*sn*‐glycero‐3‐phosphocholine (DOPC) lipids. BCP2 (or BCP2^f^) was directly dissolved in 5 mg DOPC lipids in chloroform to overcome its solubility issues. BCP2^f^ and 1,2‐dimyristoyl‐*sn*‐glycero‐3‐phosphoethanolamine‐N‐(lissamine rhodamine B sulfonyl) (^Rhod^PE) were used to visualize the integration of both building blocks in the same GHV2 by spinning disk confocal microscopy (SDCM). The assembled GHVs will be referred to as GHVX^n^ or ^L^GHVX^n^ where X refers to the type of block copolymer and n to the mol% of BCPX or BCPX^f^ (2.5, 5, or 10 in mol%), while L indicates the type of fluorophores conjugated to the building blocks (Rhod for the lipids and NBD for the BCPX^f^). Microscopy images revealed that ^Rhod^GHV1^2.5^ was successfully assembled although aggregates were present in the bilayer (Figure S3a, Supporting Information). However, GHV1^5^ failed to form, indicating that BCP1 had limited compatibility with cDICE, which contrasts with the formation of GHVs containing 5 mol% BCP1 using electroformation.^[^ ^17^ ^]^ On the other hand, ^Rhod^GHV2^2.5^ and ^Rhod^GHV2^5^ were successfully assembled, but ^Rhod^GHV2^10^ was severely aggregated (Figure S3b, Supporting Information). We speculate that the low solubility of BCP2 in the lipid mixture in chloroform might account for this observation.

**Figure 2:**
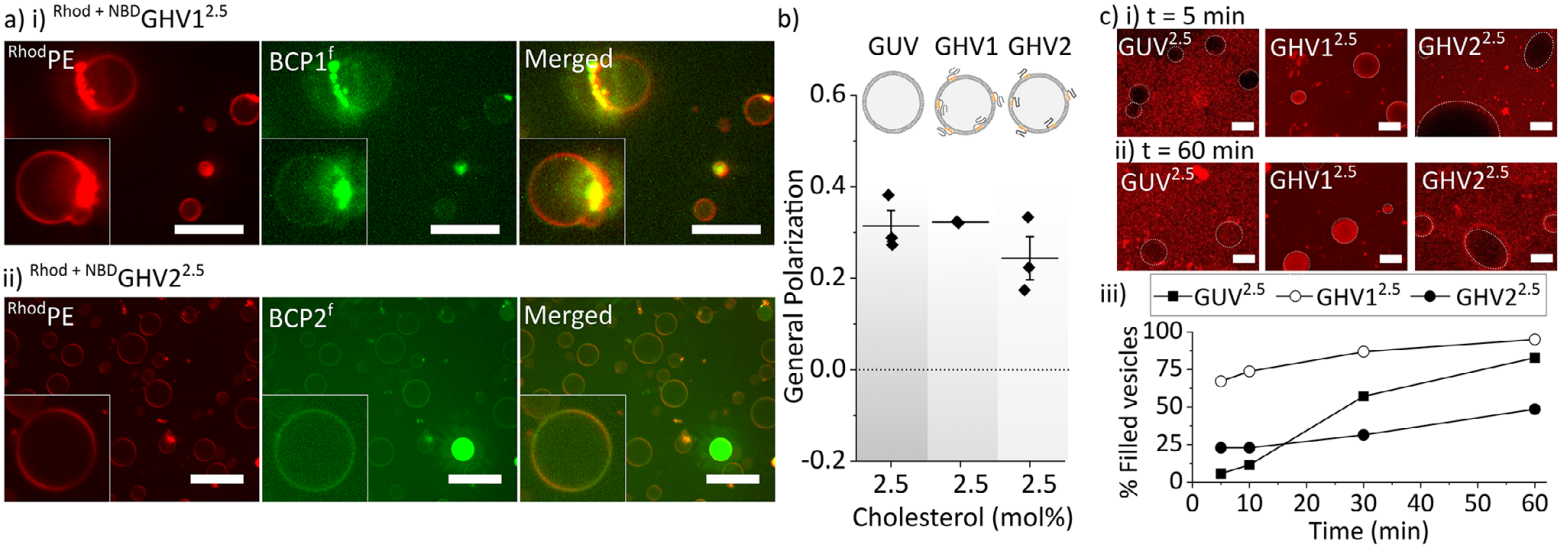
GHV characterization. a) Representative SDCM images of (i) ^Rhod+NBD^GHV1^2.5^ and (ii) ^Rhod+NBD^GHV2^2.5^ (green: BCPX^f^, red: ^Rhod^PE; Scale bar: 50 µm). b) General polarization values derived from Laurdan spectra of GUV^2.5^, GHV1^2.5^ and GHV2^2.5^. The data are expressed as mean ± SD (n = 2–3). c) i) Representative CLSM of GUV^2.5^, GHV1^2.5^ and GHV2^2.5^ after (i) 5 and (ii) 60 min incubated with 5(6)‐ROX (5 µM). (Red: 5(6)‐ROX; Scale bar: 10 µm). iii) Percentage of 5(6)‐ROX‐filled vesicles as a function of time (N = 35–60).

The fluorescence signal of both blocks was homogeneously distributed in the membrane of ^Rhod+NBD^GHV1^2.5^ and ^Rhod+NBD^GHV2^2.5^, i.e., no phase separation was observed and no BCP2^f^ was encapsulated in the form of micelles or other small aggregates in the lumen (**Figure** **2**a).

Next, we compared the membrane fluidity of GUVs composed of DOPC and the same amount of molecular cholesterol (GUV^2.5^) as present in GHVX^2.5^ in the polymerized form using the Laurdan probe (Figure 2b). The general polarization (GP) is a measure of the packing and fluidity of the membrane. The GP was calculated as GP = (I_440_ – I_490_)/(I_440_ + I_490_), where I_440_ and I_490_ represent the emission intensities at 440 and 490 nm, respectively. GHV2^2.5^ had a slightly lower GP value compared to GHV1^2.5^ and GUV1^2.5^, pointing toward differences in the membranes (Figure 2b). It should be noted that the measured GP values here (≈0.3) were vastly different compared to those of DOPC GUVs made by electroformation, where the GP values of pristine DOPC GUVs were ≈‐0.35.^[^ ^20^ ^]^ We attributed the unexpected results to the fact that these GUVs were made via the cDICE method, and the membranes may contain trace amounts of oil from the assembly process.

With the aim of getting further insight, we correlated the membrane permeability toward small molecules to the membrane composition by using a small hydrophilic molecule, 5(6)‐carboxy‐X‐rhodamine (5(6)‐ROX), which is a stable long wavelength rhodamine dye, ideal for fluorescence microscopy. We used this model compound to probe its ability to cross the membranes of GHVX^2.5^ in comparison to GUV^2.5^ (Figure 2c). The vesicles were immersed in a 5(6)‐ROX solution, and confocal scanning laser microscopy (CLSM) images were recorded for 60 min. The number of empty and filled vesicles was counted and the results were expressed as a percentage of filled vesicles. GUV^2.5^ and GHV1^2.5^ showed high permeability with ≈75% of vesicles filled after 60 min. In contrast, GHV2^2.5^ was less permeable with only ≈50% 5(6)‐ROX‐filled vesicles within this period, suggesting the incorporation of BCP2 resulted in more impermeable membranes.

### 2.3 Microtubule Polymerization in Vesicles

Microtubule polymerization involves the addition of GTP‐bound tubulin dimers to the growing plus end of the microtubule, stabilizing its structure. Once incorporated, the GTP on the β‐tubulin subunit is hydrolyzed, which weakens the tubulin binding and leads to instability unless the GTP cap (made of tubulin dimers that have GTP bound to the β‐tubulin subunit) remains intact.^[^ ^21^ ^]^ In contrast, GMPCPP is a slowly hydrolysable analog of GTP,^[^ ^22^ ^]^ which prevents the usual destabilization that occurs upon GTP hydrolysis, making GMPCPP useful for stabilizing microtubule structures. Additionally, it has been shown that the GMPCPP‐microtubules are stiffer than the GTP‐microtubules.^[^ ^23^ ^]^ Importantly, it has been demonstrated that nucleation occurred more rapidly when GMPCPP was used instead of GTP because small initial nucleation events remained stable.^[^ ^24^ ^]^ As a result, more microtubules grew simultaneously, leading to shorter microtubules compared to conditions where GTP was present. In addition, it has been previously reported that the nucleation and growth of filaments were driven by tubulin concentration (in excess of GMPCPP), i.e., higher concentrations of tubulin resulted in shorter filaments.^[^ ^25^ ^]^

**Figure 3:**
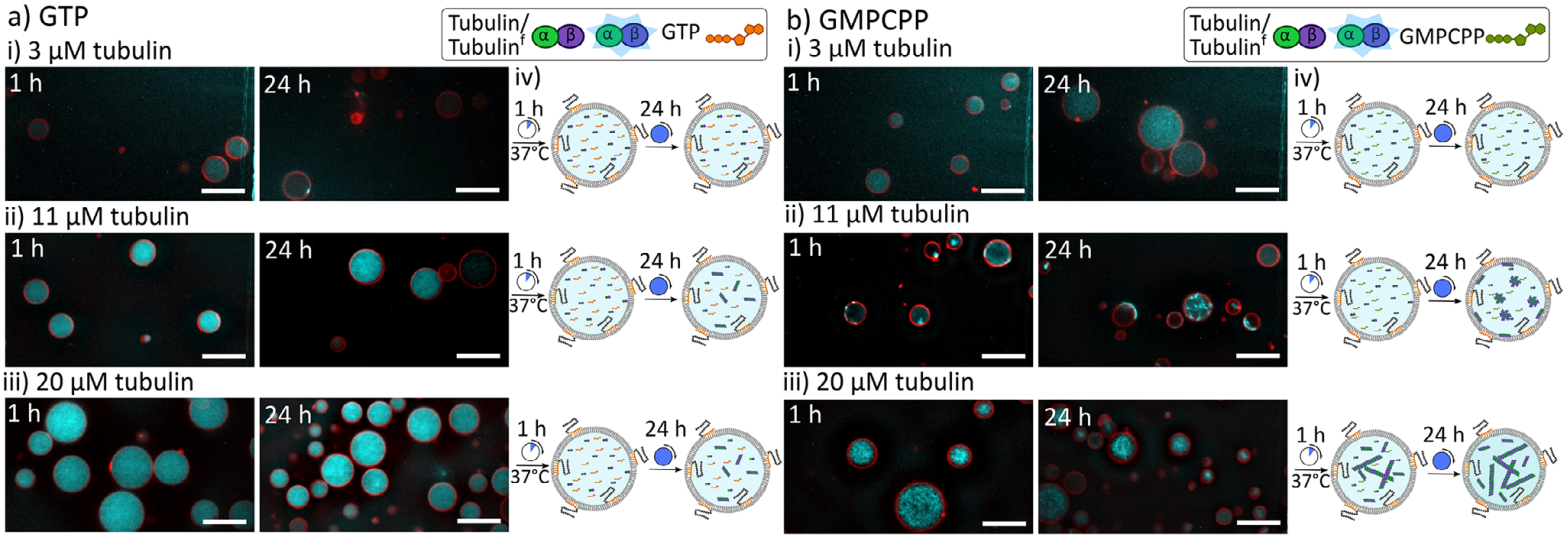
Representative SDCM images of ^Rhod^GHV2^2.5^ encapsulated with different concentrations of tubulin with 1 mm GTP (a) or 1 mm GMPCPP (b). The concentrations of tubulin were 3 µm (i), 11 µm (ii), and 20 µm (iii) (blue: tubulin^F^, red: ^Rhod^PE; Scale bar: 10 µm; n = 3). iv) Cartoon illustrating the time‐dependent, encapsulated microtubule formation.

Our first goal was to determine the optimal conditions for in situ tubulin polymerization within confined environments. For this purpose, we encapsulated increasing amounts of tubulin, supplemented with 10 wt.% fluorescently labeled tubulin (tubulin^F^), together with either GTP or GMPCPP into ^Rhod^GHV2^2.5^. We selected ^Rhod^GHV2^2.5^ for this purpose since it contained the highest amount of BCP2 without showing aggregation. SDCM images were recorded after 1 and 24 h of tubulin polymerization to visualize the early‐stage and late‐stage microtubules after polymerization, respectively. It should be noted that only the first hour of incubation was at 37 °C, while the subsequent 23 h of incubation was at room temperature. Fluorescence microscopy images did not reveal the presence of microtubules in the presence of GTP, regardless of polymerization time or tubulin concentration (**Figure** **3**a). The fluorescence intensity increases with increasing amounts of encapsulated tubulin^F^, as expected. We attributed this observation to the fast GTP hydrolysis rate compared to microtubule growth rate, leading to catastrophe (depolymerization of the microtubules) due to the loss of the GTP cap. However, since we did not observe microtubules in this case, we cannot exclude the possibility that either no tubulin polymerization occurred, or that tubulin polymerized but failed to form stable microtubules.

In contrast, when GMPCPP was used instead of GTP, microtubules were observed after 1 and 24 h of incubation at tubulin concentrations of 11 and 20 µm, especially after 24 h (Figure 3b). However, we did not observe microtubule polymerization at 3 µm of tubulin, which is below the critical concentration for polymerization (Figure 3b (i)). Notably, the location and morphology of the microtubules were dependent on the concentration of tubulin, as the GMPCPP concentration was maintained at a constant 1 mm throughout the experiments. Encapsulation of a lower concentration of tubulin (11 µm; GMPCPP:tubulin ratio ≈90:1) led to the formation of partially membrane‐associated microtubules after 1 h of polymerization. These membrane‐associated structures became more pronounced over the following 23 h, accompanied by the appearance of microtubule aggregates or clusters within the lumen of ^Rhod^GHV2^2.5^ probably due to the hydrolysis of GMPCCP and the subsequent destabilization of the microtubules (Figure 3b (ii)). In contrast, the higher amount of tubulin (20 µm; GMPCPP:tubulin ratio ≈50:1) led to the formation of a microtubule network in the lumen of ^Rhod^GHV2^2.5^ within 1 h, without noticeable membrane association. The microtubule network showed densification in the lumen of ^Rhod^GHV2^2.5^ in the subsequent 23 h of incubation (Figure 3b (iii)). This observation was likely due to the tubulin concentration‐dependent filament growth. The lower tubulin concentration (11 µm) resulted in longer microtubules that buckled on the edge of the ^Rhod^GHV2^2.5^ whereas the higher tubulin concentration (20 µm) led to shorter filaments that assembled in a mesh/network in the lumen of ^Rhod^GHV2^2.5^. Since microtubule networks more closely mimicked biological systems, we decided on a tubulin concentration of 20 µm and using GMPCPP for encapsulation in ^Rhod^GHV2^2.5^. This allowed us to further assess microtubule formation across varying GMPCPP concentrations and with different membrane compositions.

**Figure 4:**
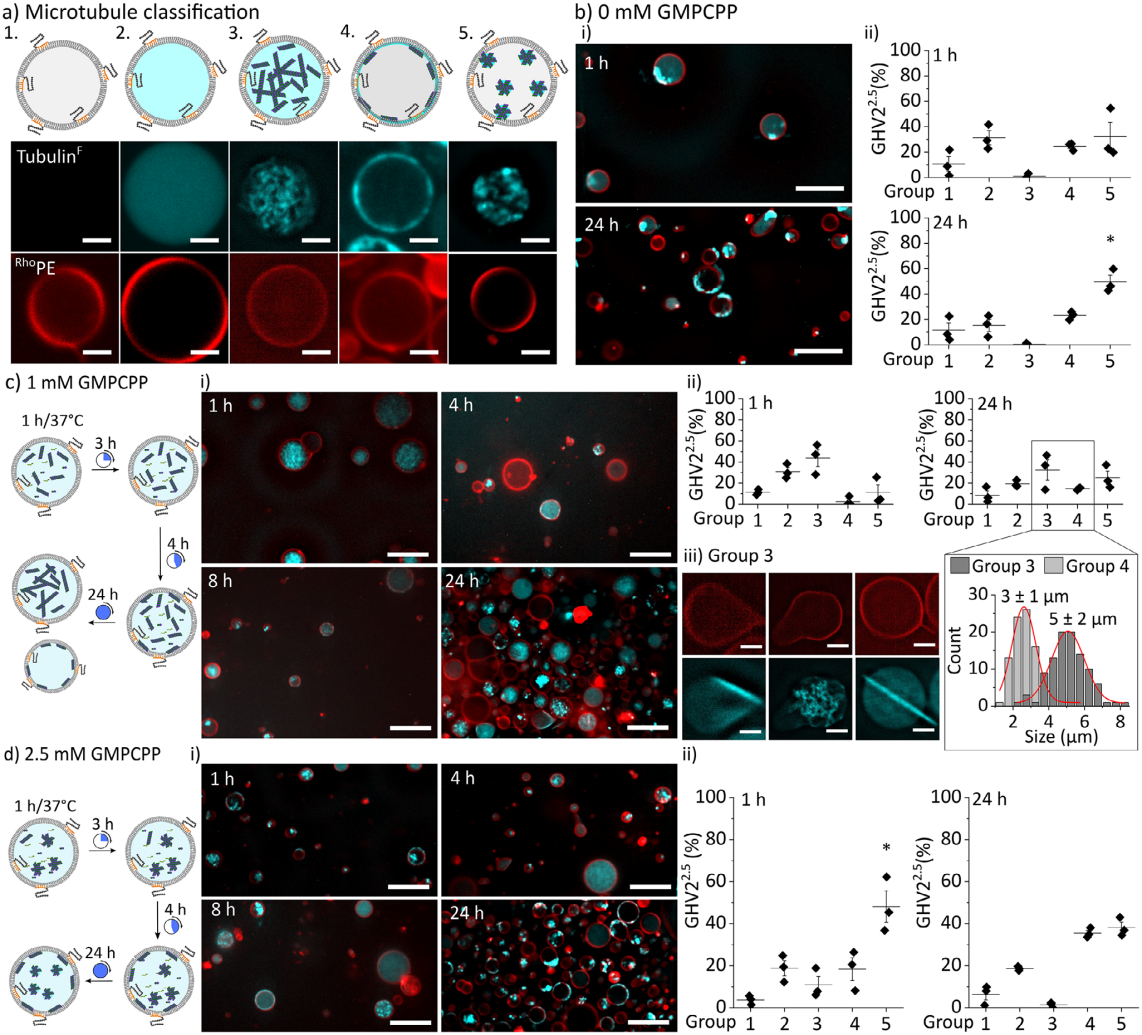
a) Representative schematics and SDCM images of the different phenotypes of tubulin‐encapsulating ^Rhod^GHV2^2.5^ (Scale bar: 5 µm). ^Rhod^GHV2^2.5^ were loaded with either b) 0 mm, c) 1 mm, or d) 2.5 mm GMPCPP together with 20 µm tubulin, and they were observed over 24 h. (i) Representative images after 1, 4, 8 and 24 h (Scale bar: 10 µm). (ii) The distribution of the different morphologies in the population after 1 and 24 h. At least 100 vesicles were quantified per experiment (n = 3, ^*^ *p* < 0.05, one‐way ANOVA with Šídák's multiple‐comparisons test). c) ii ‐ inset) Representative histogram of vesicle diameters for Group 3 and 5, measured from images of samples incubated for 24 h. A minimum of 100 vesicles were measured for each group, and the resulting histograms were fitted to a Gaussian amplitude function, yielding the mean size and variance (given as mean diameter ± 2σ) as indicated above the histograms. c) iii) Representative super‐resolution images of GHV2^2.5^ in Group 3 after 24 h incubation with 1 mm GMPCPP showing different types of deformed GHV2^2.5^ (red: ^Rhod^PE, cyan: tubulin^F^; n = 3; Scale bar: 5 µm).

To this end, we encapsulated 20 µm tubulin (with 10 w% tubulin^F^) together with either 1 or 2.5 mm GMPCPP in ^Rhod^GHV2^2.5^ and recorded confocal fluorescence images after 1, 4, 8, and 24 h incubation. In general, microtubules were observed in all cases, however, there was notable variation in the morphology of microtubules across individual ^Rhod^GHV2^2.5^. Therefore, we classified individual ^Rhod^GHV2^2.5^ into different groups (**Figure** **4**a). Empty ^Rhod^GHV2^2.5^ and ^Rhod^GHV2^2.5^ with non‐polymerized tubulin were Group 1 and Group 2, respectively. Group 3 contained ^Rhod^GHV2^2.5^ where the microtubules formed a network‐like morphology in the lumen and were membrane‐associated, while Group 4 consisted of ^Rhod^GHV2^2.5^ with membrane‐associated microtubules without fluorescence signal originating from tubulin^F^ in the lumen. Group 5 was defined as ^Rhod^GHV2^2.5^ where the fluorescence signal originated from a clump of tubulin^F^‐containing material that did not appear to be a network of microtubules.

To evaluate if microtubules could spontaneously form in ^Rhod^GHV2^2.5^, we encapsulated only tubulin in the absence of any nucleotide or GMPCPP. We found that after 1 h incubation, some ^Rhod^GHV2^2.5^ exhibited a homogenous fluorescence signal in their lumen (Group 2), others from their membranes (Group 4) while a similar fraction of ^Rhod^GHV2^2.5^ contained aggregates (Group 5). After 24 h incubation, ^Rhod^GHV2^2.5^ mostly contained tubulin^F^ clumps (Group 5) (Figure 4b). However, it was not possible to discriminate from the fluorescence images if tubulin^F^ associated with the membrane or if microtubules were formed in this case, although the absence of GMPCPP made microtubule polymerization unlikely.

Next, we encapsulated tubulin and 1 mm GMPCPP in ^Rhod^GHV2^2.5^ (Figure 4c (i)). We found that less than 10% of the ^Rhod^GHV2^2.5^ had no tubulin fluorescence (Group 1), illustrating relatively homogenous encapsulation of tubulin^F^. Group 3 was the dominating phenotype (≈50% at 1 h) when using 1 mm GMPCPP for 1 and 24 h incubation, illustrating successful intravesicular microtubule network formation (Figure 4c (ii)). Further, the abundance of Group 5 increased between 1 and 24 h, likely due to the hydrolysis of the GMPCPP that led to depolymerization, shown as time‐dependent aggregation of the microtubules in the lumen of the ^Rhod^GHV2^2.5^. The population of Group 4 slightly increased over time, but the fraction of ^Rhod^GHV2^2.5^ with membrane‐associated microtubules remained low. Additionally, after 24 h, ≈20% of all ^Rhod^GHV2^2.5^ (Group 2) had encapsulated tubulin, but we could not detect any filamentous microtubules within vesicles. Moreover, a ^Rhod^GHV2^2.5^ size‐dependent trend was observed when the diameters of ^Rhod^GHV2^2.5^ in Groups 3 and 4 were analyzed (Figure 4c (ii)). Results suggested that larger ^Rhod^GHV2^2.5^ (≈5 µm) mostly showed filamentous microtubules (Group 3) whereas smaller ^Rhod^GHV2^2.5^ (≈3 µm) contained membrane‐associated microtubules (Group 4). Although other factors than spatial constraints might play a role in microtubule polymerization, we hypothesized that the physical boundary of ^Rhod^GHV2^2.5^ explained the observed morphologies. Smaller ^Rhod^GHV2^2.5^ had lower internal volume and higher curvature that restricted the ability of microtubules to grow freely, causing the microtubules to bend, and leading to their buckling at the edges of vesicles.

Interestingly, ≈10% of the ^Rhod^GHV2^2.5^ in Group 3 were deformed in a variety of morphologies after 24 h incubation (Figure 4c (iii)). Midzone (central spindle) assembly of the microtubules resulted in elliptic deformation of ^Rhod^GHV2^2.5^. In addition, some microtubule networks seemed to be partly associated with the membrane, inducing deformation. In the latter case, most of these ^Rhod^GHV2^2.5^ had a microtubule network present in their lumen, but a small fraction had only a portion of their membrane associated with microtubules with non‐polymerized tubulin in the lumen. Although we currently cannot explain these observations in detail, they give insight into the complexity of microtubule formation in giant vesicles.

Increasing the amount of GMPCPP from 1  to 2.5 mm affected the morphologies of encapsulated microtubules (Figure 4d (i)). While Group 5 had the highest abundance after 1 h incubation, Group 4 and 5 were comparable after 24 h incubation (Figure 4d (ii)). We attributed this finding to the fast microtubule polymerization due to the high amount of GMPCPP‐bound tubulin dimers (GMPCPP:tubulin≈125:1). It should be noted that this membrane‐bound cortex‐like morphology (Group 4) has been previously reported using membrane‐anchored proteins to mediate membrane attachment.^[^ ^15^ ^]^ In contrast, the membrane‐associated microtubules we observed were in the absence of any microtubule‐associated proteins, suggesting our polymer‐lipid may play a role here. We note that tubulin was found to lower interfacial surface tension at the oil‐water interface in a lipid‐oil dispersion, potentially accounting for the observation of membrane‐associated microtubules.^[^ ^26^ ^]^

**Figure 5:**
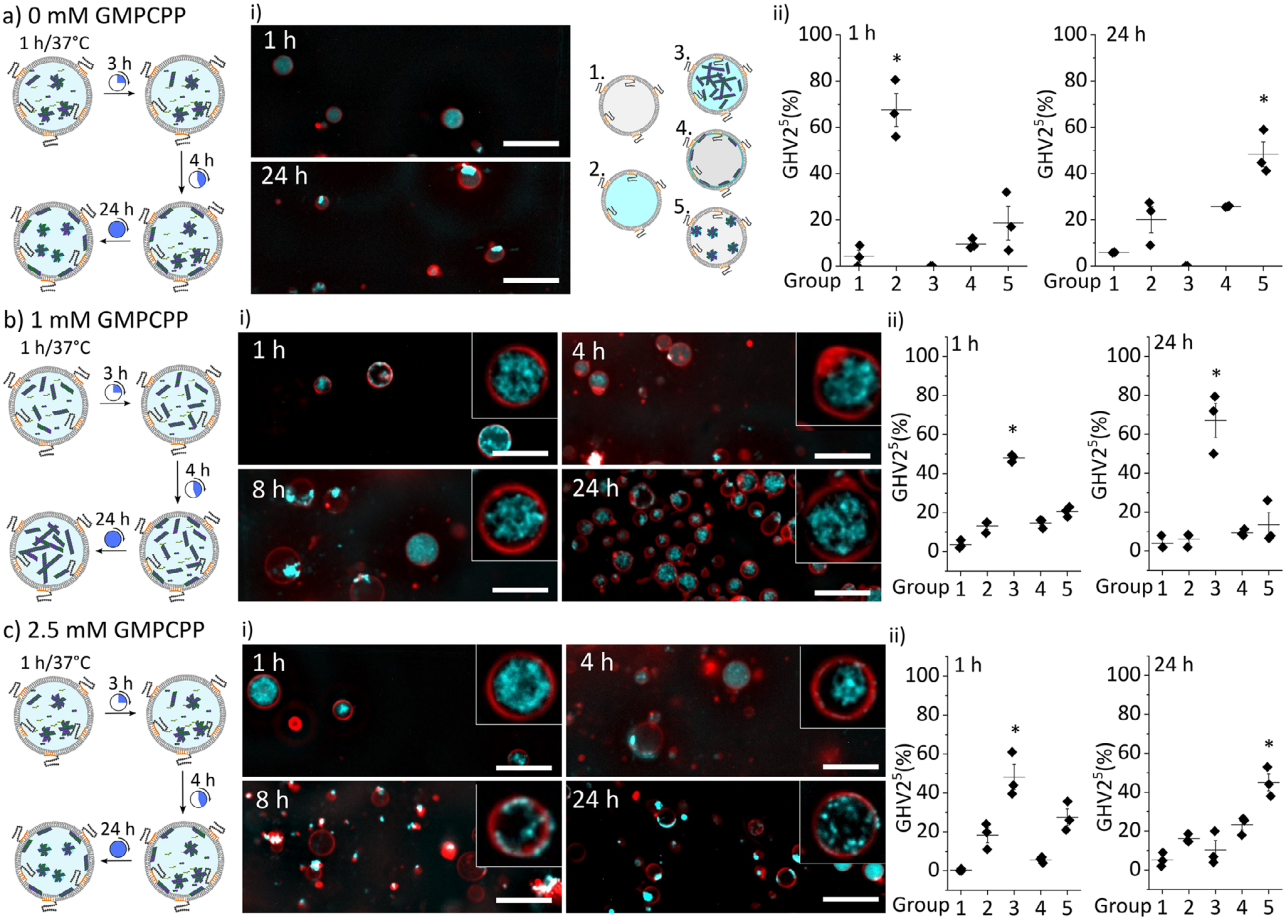
^Rhod^GHV2^5^ encapsulated with either a) 0 mm, b) 1 mm, or c) 2.5 mm GMPCPP together with 20 µm tubulin were observed over 24 h. (i) Representative confocal microscopy images after 1, 4, 8, and 24 h (red: ^Rhod^PE, cyan: tubulin^F^; n = 3; Scale bar: 10 µm). (ii) The distribution of the different morphologies in the population after 1 and 24 h. At least 100 vesicles were quantified per experiment (n = 3, ^*^ *p* < 0.05, one‐way ANOVA with Šídák's multiple‐comparisons test).

Previous studies suggested that hydrophilic molecules such as polyethylene glycol affects nucleation and growth rate, leading to filament contour length changes.^[^ ^27^ ^]^ Since the sugar‐like units present in the block copolymer were also hydrophilic, we explored the impact of the BCP2 on the encapsulated tubulin polymerization. Specifically, ^Rhod^GHV2^5^ (vesicles made with double the amount of BCP2 compared to ^Rhod^GHV2^2.5^) were employed and 20 µm tubulin (with 10 w% tubulin^F^) were encapsulated (**Figure** **5**). First, we evaluated the resulting microtubule morphologies in the absence of GMPCPP. After 1 h incubation, most of the ^Rhod^GHV2^5^ exhibited a homogenous fluorescence signal in their lumen (Group 2) whereas, after 24 h, a greater number of ^Rhod^GHV2^5^ contained tubulin clumps (Group 5), which was similar to the results observed for ^Rhod^GHV2^2.5^ (Figure 5a). Further, when 1 mm GMPPCP was employed, a significantly higher number of ^Rhod^GHV2^5^ containing microtubule filaments (Group 3) was observed both after 1 and 24 h incubation (Figure 5b). Interestingly, most of the ^Rhod^GHV2^5^ exhibited spherical shapes with no apparent deformations. These findings differed from the results observed for ^Rhod^GHV2^2.5^, where different morphologies were present in the population and deformations were observed after 1 h.

Further, increasing GMPCPP to 2.5 mm again led to the most ^Rhod^GHV2^5^ belonging in Group 3 after 1 h incubation (Figure 5c). In addition, aggregated microtubules (Group 5) in ^Rhod^GHV2^5^ dominated after 24 h incubation. These observations contrasted the outcome when ^Rhod^GHV2^2.5^ was used (Figure 4c,d). The only difference between ^Rhod^GHV2^2.5^ and ^Rhod^GHV2^5^ was the amount of BCP2 used, which strongly suggested that the presence of the hydrophilic extensions affected microtubule polymerization. Overall, the presence of a higher concentration of hydrophilic polymer appeared to promote the formation of a stable microtubule network, in particular for lower GMPCPP concentrations, maintaining its structural integrity over time.

This initial finding offers interesting future opportunities for using synthetic membranes for controlling the architecture of artificial cytoskeleton formation.

To assess whether membrane composition played a role in the observed microtubule morphologies, we encapsulated 20 µM tubulin and GMPCPP in either ^Rhod^GUV^2.5^ or ^Rhod^GHV1^2.5^. We observed tubulin^F^ fluorescence at the membrane of ^Rhod^GUV^2.5^, and no lumenal microtubule network after 1 h incubation irrespective of GMPCPP concentration (Figure S4, Supporting Information). Additionally, microtubules were observed outside the ^Rhod^GUV^2.5^ after 24 h incubation. This observation might be explained by either leakage of the tubulin and/or GMPCPP, rupture of ^Rhod^GUV^2.5^ due to microtubule formation, or tubulin and/or GMPCPP not encapsulated as well as a combination thereof. These results were consistent with the data from the 5(6)‐ROX permeability assay, which showed that GUV^2.5^ was more permeable than GHV2^2.5^, which could have affected the encapsulation process and the subsequent cargo retention. We would like to note that alternative assembly protocols and other membrane compositions were successful for in situ microtubule formation in GUV.^[^ ^11^, ^12^, ^13^, ^14^, ^15^ ^]^ However, we wanted to compare membranes that contained the same amount of either polymerized cholesterol units or molecular cholesterol using the same giant vesicle assembly protocol.

Furthermore, ^Rhod^GHV1^2.5^ was used to assess if the type of hydrophilic extension in the amphiphilic block copolymer influenced the microtubule morphologies. It should be noted that a low yield of ^Rhod^GHV1^2.5^ was obtained using cDICE. This was likely due to the higher amphiphilicity for BCP2 compared to BCP1. Similar to ^Rhod^GUV^2.5^, no microtubule networks were observed in the lumen of ^Rhod^GHV1^2.5^, and only membrane‐associated tubulin/microtubules were present after 1 h incubation. Moreover, no significant changes were observed after 24 h incubation (Figure S5, Supporting Information). These results were consistent with the data from the 5(6)‐ROX permeability assay, which showed that GHV1^2.5^ was significantly more permeable than GHV2^2.5^. Taken together, these results suggest that the membrane composition of the vesicles plays a critical role in microtubule formation and organization.

## 3 Conclusion

We report the in situ formation of microtubule networks within giant polymer‐lipid hybrid vesicles ^Rhod^GHV2^2.5^ and ^Rhod^GHV2^5^ in the presence of GMPCPP. Interestingly, we found distinct microtubule morphologies as a function of time and GMPCPP concentration. Overall, our findings illustrate the potential of using different membrane compositions to alter encapsulated microtubule morphologies. Future efforts will focus on increasing the complexity of the cytoskeleton including its reversible assembly and disassembly as well as its interaction with relevant membrane proteins. Integration with motor and membrane proteins could further enhance functionality, mimicking natural cellular processes such as intracellular transport and signaling. These advancements provide a foundation for exploring fundamental biophysical principles and pave the way for engineering life‐like artificial cells with enhanced functionality.

## 4 Experimental Section

### Materials

1,2:3,4‐Di‐O‐isopropylidine‐α‐galactopyranose (proGalac), methacryloyl chloride, butyl methacrylate (BuMA), 2‐(dodecylthiocarbonothioylthio)‐2‐methylpropionic acid, N‐hydroxysuccinimide ester (NHS‐CTA, 98%), triethylamine (Et_3_N), PMMA standard (Mn ≈ 104 000 Da), 2,2′‐azobis(2‐methylpropionitrile) (AIBN, recrystallized), toluene, silicone oil, 6‐dodecanoyl‐N,Ndimethyl‐2‐naphtylamine (Laurdan), calcein, glucose, magnesium chloride (MgCl_2_), 1,4‐piperazinediethanesulfonate (PIPES), sodium carbonate, 5(6)‐carboxy‐X‐rhodamine (5(6)‐ROX) (λ_ex_/λ_em_ = 578/604 nm) and ethylene glycol‐bis(β‐aminoethyl ether)‐N,N,N,N‐tetraacetic acid (EGTA) were purchased from Sigma Aldrich. Mineral oil pure (415 080 010), guanosine triphosphate (GTP), and dithiothreitol (DTT) were purchased from Thermo Fisher Scientific. Chloroform (CHCl_3_), chloroform‐d (CDCl_3_, 99.80% D), ethyl acetate (EtOAc), acetonitrile, methanol (MeOH), tetrahydrofuran anhydrous (THF), magnesium sulfate, hexane, pyridine‐d5, and acetone were purchased from VWR. 1,2‐Dioleoyl‐*sn*‐glycero‐3‐phosphocholine (DOPC) and 1,2‐dimyristoyl‐*sn*‐glycero‐3‐phosphoethanolamine‐N‐(lissamine rhodamine B sulfonyl) (ammonium salt) (^Rhod^PE) were purchased from Avanti Polar Lipids. Cycled tubulin and labeled tubulin‐Alexa Fluor 647 were purchased from PurSolutions. GMPCPP was purchased from Jena Biosciences. Dialysis tubing (MWCO 3.5 kDa, Spectra/POR 3) was obtained from Spectrumlabs. 4‐(N‐chloroformylmethyl‐N‐methylamino)‐7‐nitro‐2,1,3‐benzoxadiazole (NBD‐COCl) and trifluoroacetic acid (TFA) were purchased from TCI.

Ultrapure water (18.2 MΩ cm resistance) was provided by Synergy UV by Millipore.

Poly(cholesteryl methacrylate‐*co*‐2‐hydroxyethyl methacrylate)‐*co*‐butyl methacrylate)‐*block*‐poly(2‐carboxyethyl acrylate) (BCP1), its fluorescent version BCP1^f^, and poly(cholesteryl methacrylate‐*co*‐butyl methacrylate) (pCMA‐*co*‐BuMA) were synthesized as previously described.^[^ ^17^ ^]^ Cholesteryl methacrylate (CMA) was synthesized as previously described.^[^ ^28^ ^]^

BRB80 buffer consisted of 80 mm PIPES, 1 mm MgCl_2,_ and 1 mm EGTA (pH 6.8).

### Synthesis of 6‐O‐Methacryloyl‐1,2:3,4‐di‐O‐Isopropylidene‐α‐Galactopyranose Monomer (proGalacMA)

ProGalacMA was synthesized according to previously published procedures.^[^ ^18^ ^]^ Specifically, proGalac (1 g, 3.8 mmol) was dissolved in 15 mL acetonitrile before the addition of Et_3_N (1.2 mL, 5.8 mmol). Methacryloyl chloride (0.56 mL, 3.8 mmol) was added dropwise to the solution, and it was left to stir overnight at room temperature. The reaction mixture was filtered and dried by rotary evaporation. The remaining oily substance was then redissolved into 30 mL chloroform and washed with saturated Na_2_CO_3_ (5×100 mL) followed by ultrapure water (5×100 mL). The organic layer was then dried over magnesium sulfate. The obtained solution was cleaned through column chromatography (ethyl acetate/hexane, 1:3, v/v). The fractions containing the product were combined, and the solvent was removed by rotary evaporation. The product was left in the fridge overnight, where it solidified, resulting in a white powder. ^1^H NMR of crude and purified products were taken on a Bruker Ascend 400. MestReNova software was used to analyze the spectra. The monomer synthesis was summarized in Scheme S1 (Supporting Information).

^1^H NMR (400 MHz, CDCl_3_) δ (ppm): 6.13 (dt, J = 2.0, 1.0 Hz, 1H), 5.57 (p, J = 1.6 Hz, 1H), 5.54 (d, J = 4.9 Hz, 1H), 4.63 (dd, J = 7.9, 2.5 Hz, 1H), 4.39 – 4.22 (m, 2H), 4.07 (ddd, J = 7.1, 4.8, 1.9 Hz, 1H), 1.95 (dd, J = 1.6, 1.0 Hz, 3H), 1.51 (s, 3H), 1.46 (s, 3H), 1.34 (d, J = 4.5 Hz, 7H).

### Synthesis of Poly(Cholesteryl Methacrylate ‐co‐ Butyl Methacrylate) – block – Poly (Galacto Methacrylate) (p(CMA‐co‐BuMA)‐b‐p(proGalacMA))

pCMA‐*co*‐BuMA (150 mg), proGalacMA (331 mg) and AIBN (0.6 mg) were mixed and dissolved in 1.8 mL toluene. The flask was bubbled with argon for at least 30 min, and subsequently transferred to an oil bath (74 °C) and stirred overnight. The resulting polymer was cleaned through precipitation into 20 mL MeOH and left to dry. A white powder was obtained referred to as BCP2^p^. The polymerization synthesis was summarized in Scheme S2 (Supporting Information).

^1^H NMR (400 MHz, CDCl_3_) δ (ppm): 5.51 (s, 33H), 5.35 (s, 9H), 4.64 (s, 30H), 4.48 (s, 8H), 4.32 – 3.89 (m, 201H), 2.80 (s, 4H), 2.37 – 0.54 (m, 767H).

### Deprotection of BCP2^p^ (p(CMA‐co‐BuMA)‐b‐p(GalacMA))

BCP2^p^ (350 mg) was dissolved in 10 mL TFA and stirred overnight. The polymer was dried through rotary evaporation, redissolved in THF, and dialyzed (MWCO 3.5 kDa) against ultrapure water. Finally, the polymer was freeze‐dried and stored at ‐18 °C. A white powder was obtained referred to as BCP2.

^1^H NMR (400 MHz, Pyr) δ (ppm): 6.01 (s, 1H), 5.89 (s, 5H), 5.62 – 4.05 (m, 234H), 2.95 – 0.56 (m, 201H).

### NBD Labeling of BCP2 (BCP2^f^)

BCP2 (100 mg) was dissolved in 50 mL pyridine overnight. NBD‐COCl (10 mg) was added, and it was stirred for 48 h. The mixture was purified by dialyzing (3.5 kDa cut off) against ultrapure water. Finally, the polymer was freeze‐dried and stored at ‐18 °C. A yellow powder was obtained, but the conjugation of NBD was not detectable in ^1^H NMR, likely due to the low solubility of *BCP2^f^*.

### Gel Permeation Chromatography (GPC)

The dried polymers (≈ 10 mg) were dissolved in THF (2 mL) and filtered through a 0.2 µm PTFE filter. Experiments were carried out at 40 °C with a flow rate of 1 mL min^−1^ using THF as the mobile phase. The GPC set‐up consisted of a LC‐20AD Shimadzu HPLC pump, a Shimadzu RID‐10A refractive index detector, and a Wyatt DAWN HELEOS 8 Light scattering (LS) detector as well as a column system. A PLgel Individual Pore Size Linear column (5 µm particles, 500 Å pore size, 300 mm length, and an internal diameter of 7.5 mm) (Agilent) provided an effective molecular weight range up to 25 kDa combined with a ResiPore linear column (3 µm particles, 300 mm length and an internal diameter of 7.5 mm (Agilent)) providing an effective molecular weight range up to 500 kDa.

### Giant Unilamellar Vesicle (GUV) and Giant Hybrid Vesicle (GHV) Assembly

Encapsulation of aqueous material inside giant vesicles was achieved using a modified continuous droplet interface crossing encapsulation (cDICE) method.^[^ ^19a^ ^]^ Briefly, a 3D‐printed cDICE chamber was mounted onto a tabletop stirring motor and rotated at 1200 rpm. First, 700 µL outer aqueous glucose solution (231 mm) was dispensed into the chamber. Next, 2.1 mL oil/polymer‐lipid mixture was dispensed into the chamber. The oil/polymer‐lipid mixture composition is summarized in Table S1 (Supporting Information). The oil was composed of 75% silicon oil and 25% mineral oil. Separately, 700 µL oil/lipid mix was dispensed into a tube containing 10–20 µL prepared inner solution (encapsulant) and pipetted up and down to form an emulsion. The encapsulant composition is summarized in Table S2 (Supporting Information). Finally, the solution was transferred to the cDICE chamber where the encapsulant emulsion was shuttled through the oil/lipid mix into the outer solution. The assembled GHV were referred to as GHVX^n^ or ^L^GHVX^n^ where X refers to the type of block copolymer, n to the mol% of BCPX, and L indicates the type of fluorophores conjugated to the building blocks. The assembled GUV were referred to as GUV^m^ where m refers to the mol% of cholesterol.

### Visualization of GHVXs/GUVXs by Spinning Disk Confocal Microscopy (SDCM)

An Olympus IX‐81 inverted microscope equipped with a spinning disk confocal (Yokogawa CSU‐X1), customed solid‐state laser launch (Solamere Technology), and an iXON3 EMCCD camera (Andor Technology) was used for microscopy. Images were acquired via an oil immersion 60×/1.4 NA objective lens mounted on the microscope. The vesicles were placed in a 96‐well black/clear bottom plate, TC surface (Thermo Fisher), and the images were acquired by using MetaMorph software (Molecular Devices). Fluorescence images of BCPX^f^, ^Rhod^PE, and tubulin^F^ were taken with λ_ex_ = 488 nm, λ_ex_ = 561 nm and λ_ex_ = 640 nm, respectively.

### Visualization of GHVXs/GUVs by Confocal Laser Scanning Microscopy (CLSM)

≈200 µL vesicle solution was placed in a 96‐well black/clear bottom plate, TC surface (Thermo Fisher), and let to equilibrate for 10 min before visualization using a Zeiss LSM700 CLSM (Carl Zeiss, Germany). At least 5 images were taken from different areas for each sample. The following settings were used: 63 × magnification, images recorded with 1024 × 1024 ppi resolution, and 1 line averaging was used.

### Visualization of GHVXs by Super‐Resolution Microscopy

Approximately 200 µL vesicle solution was placed in a 96‐well black/clear bottom plate, TC surface (Thermo Fisher) and let to equilibrate for 10 min before visualization using a Nikon Yokogawa CSU‐W1 SoRa confocal. At least 5 images were taken from different areas for each sample. The following settings were used: 63 × magnification, images recorded with 1024 × 1024 ppi resolution, and 4‐line averaging was used.

### Membrane Fluidity of Giant Vesicles

The membrane fluidity of the giant vesicles was determined utilizing the fluorescent probe 6‐dodecanoyl‐N,Ndimethyl‐2‐naphtylamine (Laurdan). Here, 25 µL of the vesicle stock solution, 72.5 µL glucose (231 mm), and 2.5 µL Laurdan solution (100 µm in DMSO) were added to a black 96‐well plate and shaken at 300 rpm for at least 45 min. The emission spectrum was recorded from λ_em_ = 400–600 nm using an excitation wavelength of λ_ex_ = 340 nm.

The general polarization (GP) was determined according to the following equation.

(1) $$GP=\frac{I_{440}-I_{490}}{I_{440}+I_{490}}$$

I_440_ and I_490_ were the fluorescence intensity at λ_em_ = 440 nm and λ_em_ = 490 nm, respectively.

### 5(6)‐ROX Permeability in Giant Vesicles

A drop of 5 µm 5(6)‐ROX (5 µL) was placed on a 96 black/clear bottom plate (Thermo Fisher) and 50 µL vesicle solution was added. Images were taken every 10 min for 1 h with 63× magnification, a resolution of 1024 × 1024 ppi, and λ_ex_ = 555 nm for 5(6)‐ROX, using a Zeiss LSM700 confocal laser scanning microscope (CLSM, Carl Zeiss, Germany). Two independent repeats were made of each sample. The analysis was carried out using the ImageJ software. At least 30 vesicles were analyzed. Vesicles that had at least 50% of the highest intensity measured were considered as filled vesicles.

### Microtubule Formation in GHVs/GUVs

The encapsulation of tubulin in GUVs or GHVXs was performed as described above. The detailed encapsulant composition, i.e., GMPCPP, GTP, and tubulin, is summarized in Table S2 (Supporting Information). After assembly, the samples were incubated in a water bath at 37 °C for 1 h prior to imaging. Encapsulated vesicles were observed in a 96‐well Corning glass well plate. Then, the vesicles were kept at room temperature for 23 h and reimaged. A minimum of 5 images were taken and at least 100 vesicles per repeat were counted and three independent repeats were performed. Subsequent analysis was carried out using ImageJ software. Furthermore, the images were post‐processed using the deblurring tool (a computational deconvolution method) in Zen 3.5 (blue edition) software (Strength ≈0.7–0.9; blurRadius≈30–50). The statistical significance was determined using one‐way ANOVA with Šídák's multiple‐comparisons test, ^*^ *p *< 0.05.

The size distributions of vesicles were calculated from the microscopy images by measuring at least 100 vesicles using Fiji. A Gaussian amplitude function was used to fit the resulting histograms, and the sizes are given as the mean ± 2σ.

### Statistical Analysis

The pre‐processing of data is described in the different parts of the experimental section. The sample size (n) for each statistical analysis is 3 unless indicated otherwise. Data are presented as mean ± standard deviation. The statistical significance was determined using one‐way ANOVA with Šídák's multiple‐comparisons test, ^*^ *p* < 0.05.

## Conflict of Interest

The authors declare no conflict of interest.

## Supporting information



Supporting Information

## Data Availability

The data that support the findings of this study are openly available in Zenodo at https://doi.org/10.5281/zenodo.14796711, reference number 14796711.
