# Supplementary material for: Distinct Network Morphologies from In Situ Polymerization of Microtubules in Giant Polymer‐Lipid Hybrid Vesicles
Source: Adv Biol (Weinh). 2025 Mar 12;9(5):2400601. doi: 10.1002/adbi.202400601 (PMC12078856; doi:10.1002/adbi.202400601)
Supplement: Supplementary file 1 — Supporting Information [file ADBI-9-2400601-s001.pdf]

ADVANCED BIOLOGY

Supporting Information

for *Adv. Biology*, DOI 10.1002/adbi.202400601

Distinct Network Morphologies from In Situ Polymerization of Microtubules in Giant Polymer-Lipid Hybrid Vesicles

Paula De Dios Andres, Mousumi Akter, Cecilie Ryberg, Brigitte Städler and Allen P. Liu**

Supporting Information

Distinct network morphologies from *in-situ* polymerization of microtubules in giant polymer-lipid hybrid vesicles

Paula De Dios Andres, Mousumi Akter, Cecilie Ryberg, Brigitte Städler and Allen P. Liu**

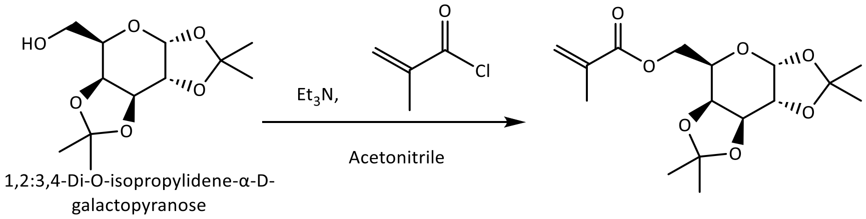

Scheme S1. Polymerization scheme of the 6-O-methacryloyl-1,2:3,4-di-O-isopropylidene-α-galactopyranose monomer (proGalacMA).

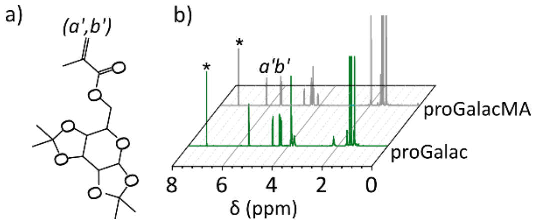

Figure S1. Structure (a) and ^{1}H NMR (b) of the proGalac and proGalacMA monomers. The ^{1}H NMR was measured in $CDCl_3$ (* indicates the solvent peak).

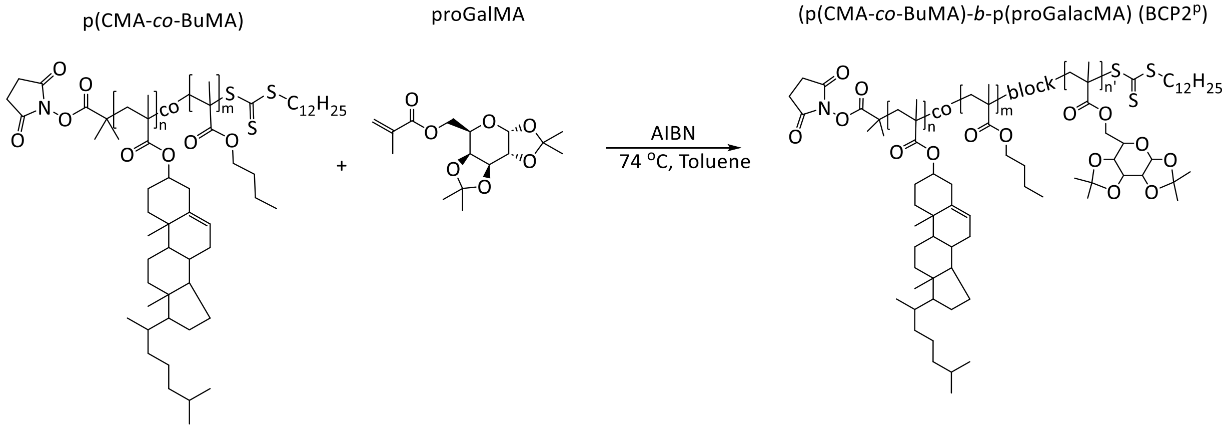

Scheme S2. Polymerization scheme of poly(cholesteryl methacrylate-*co*-butyl methacrylate)-*block*-poly(6-O-methacryloyl-D-galactopyranose) (p(CMA-*co*-BuMA)-*b*-p(proGalacMA), BCP2^P).

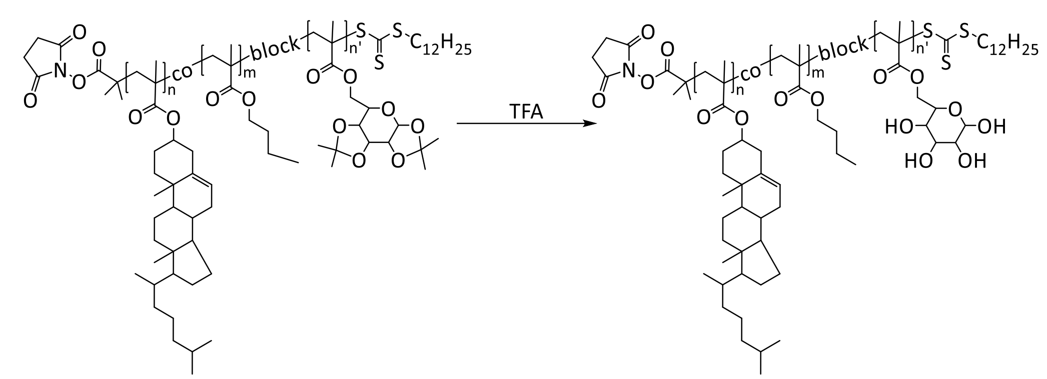

Scheme S3. Deprotection of BCP2P (p(CMA-*co*-BuMA)-*b*-p(proGalacMA)).

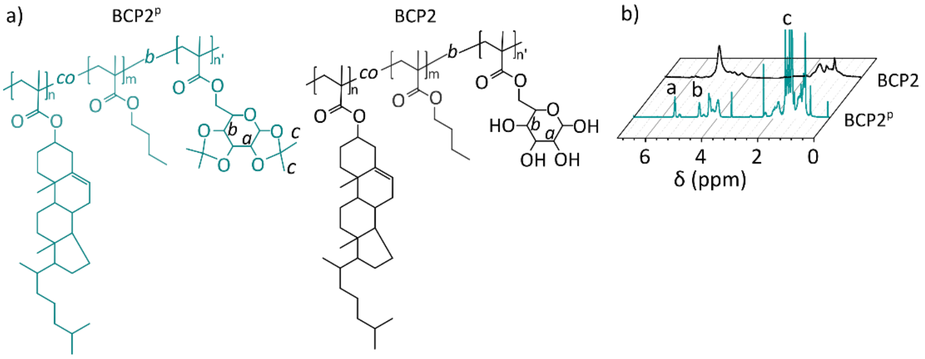

Figure S2. Structure (a) and ^{1}H NMR (b) of p(CMA-*co*-BuMA)-*b*-p(proGalacMA) (BPC2P, turquoise) in $CDCl_3$ and p(CMA-*co*-BuMA)-*b*-p(GalacMA) (BCP2, black) in pyridine *d-8*.

Table S1. Composition of the different oil/polymer-lipid mixtures.

Sample	DOPC (mg)	Cholesterol (mg)	BCP1 (mg)	BCP2 (mg)	Ratio
GUV^0	5	0.069	-	-	100:0
$GUV^{2.5}$	5	0.138	-	-	97.5:2.5
$GHV1^{2.5}$	5	-	0.65	-	97.5:2.5
$GHV1^5$	5	-	1.3	-	95:5
$GHV2^{2.5}$	5	-	-	0.65	97.5:2.5
$GHV2^5$	5	-	-	1.3	95:5
$GHV2^{10}$	5	-	-	2.6	90:10

It should be noted that, in all cases, the lipid mixture was mixed with 15 mL silicone oil and 3.8 mL mineral oil.

Table S2. Composition of the encapsulant.

		Premix						
**Sample**	**Tubulin 165 µM (µL)**	**Glucose 231 mM (µL)**	**Optiprep (µL)**	**GTP 100 mM (µL)**	**DTT 40 mM (µL)**	**GMPCPP 10 mM (µL)**	**ddH$_2$O (µL)**	**5x BRB80 buffer (µL)**
**0 mM GMPCPP**	1.5	-	0.9	-		0	8	2
**1 mM GMPCPP**	1.5	-	0.9	-		1	5	2
**2.5 mM GMPCPP**	1.5	-	0.9	-		3	7	2
**Empty vesicles**	-	18.5	1.5	-		-	-	-
**3 µM tubulin**	0.2	-	0.5	1.25	0.5	-	25.75	10
**11 µM tubulin**	0.75	-	0.5	1.25	0.5	-	25.75	10
**20 µM tubulin**	1.5	-	0.9	1.25	0.5	-	25.75	10
**3 µM tubulin**	0.2	-	0.5	-		1	7	2
**11 µM tubulin**	0.75	-	0.5	-		1	7	2
**20 µM tubulin**	1.5	-	0.9	-		1	7	2

In all cases, 9 µL of the premix was always used to mix with the indicated volume of tubulin.

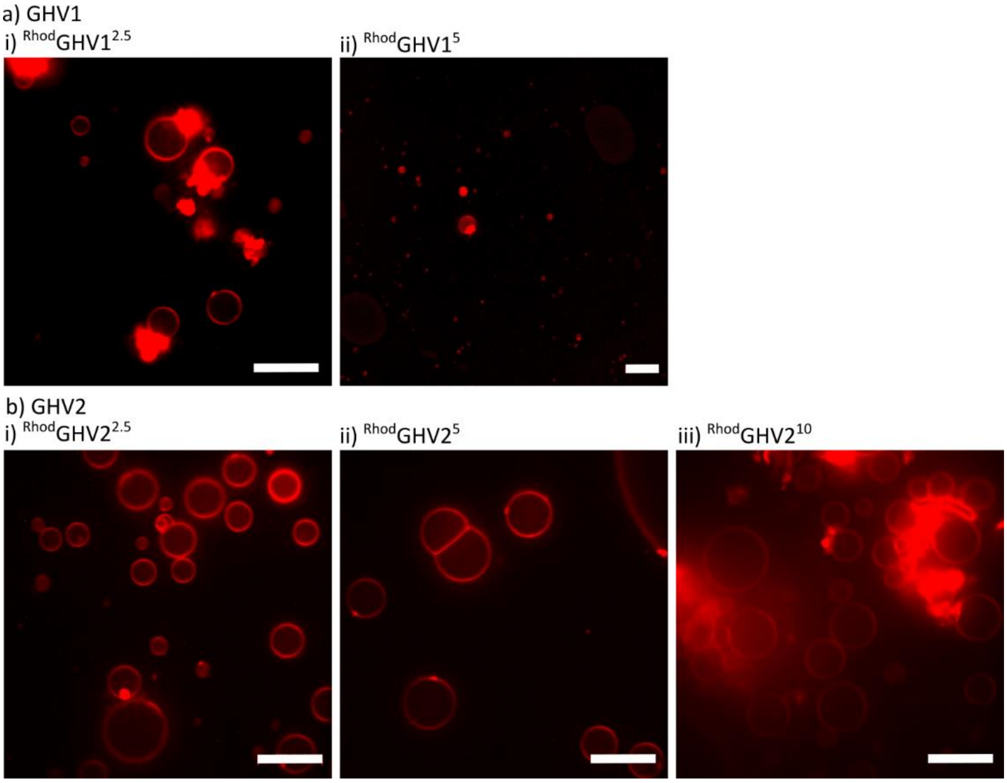

Figure S3. Representative SDCM images of $^{Rhod}GHV1^{n}$ (a) and $^{Rhod}GHV2^{n}$ (b) with 2.5% mol (i), 5% mol (ii) and 10% mol (iii) BCPX (red: ^{Rhod}PE; scale bar: 10 µm; n=3).

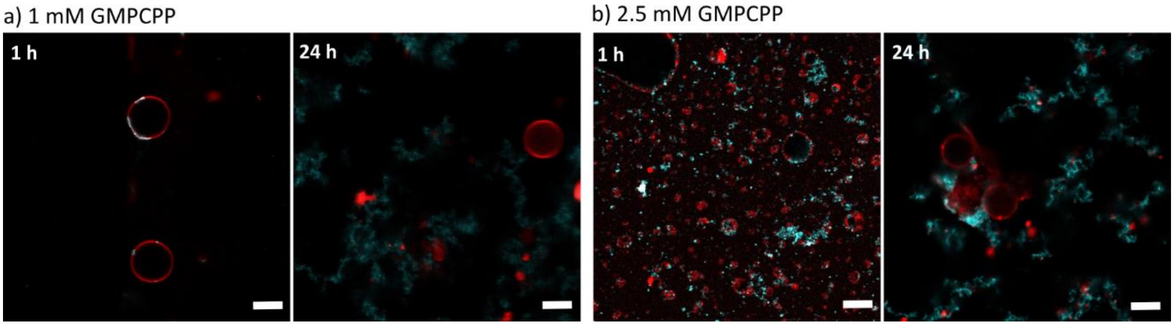

Figure S4. Representative CLSM images of $^{Rhod}GUV^{2.5}$ loaded with either 1 mM (a) or 2.5 mM (b) GMPCPP and tubulin after 1 h and 24 h incubation (red: ^{Rhod}PE, cyan: tubulin^{F}; scale bar: 10 µm; n = 3).

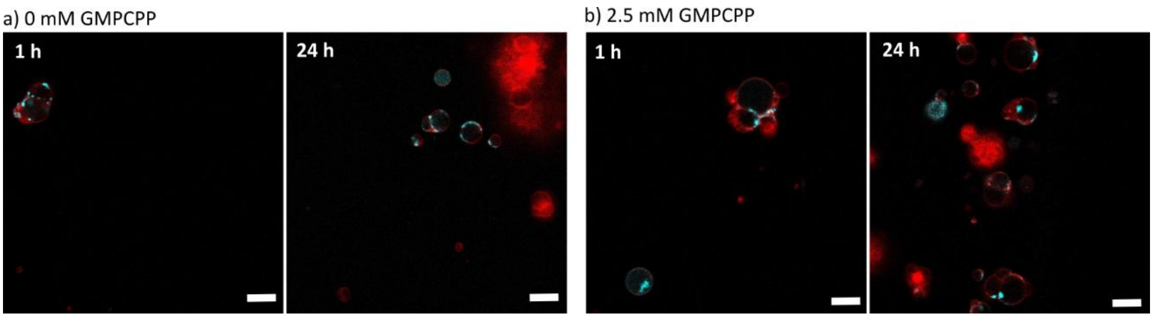

Figure S5. Representative CLSM images of $^{Rhod}GHV1^{2.5}$ loaded with either 0 mM (a) or 2.5 mM (b) GMPCPP and tubulin after 1 h and 24 h incubation (red: ^{Rhod}PE, cyan: tubulin^{F}; Scale bar: 10 µm; n = 3).
